# Hazardous waste sites and stroke in New York State

**DOI:** 10.1186/1476-069X-4-18

**Published:** 2005-08-29

**Authors:** Ivan Shcherbatykh, Xiaoyu Huang, Lawrence Lessner, David O Carpenter

**Affiliations:** 1 Department of Environmental Health and Toxicology, School of Public Health, University at Albany, SUNY, One University Place, A217, Rensselaer, NY 12144, USA; 2 Department of Biometry and Statistics, School of Public Health, University at Albany, SUNY, One University Place, A217, Rensselaer, NY 12144, USA; 3 Institute for Health and the Environment, University at Albany, SUNY, One University Place, A217, Rensselaer, NY 12144, USA; 4 McMaster University, Centre for Evaluation of Medicines, 105 Main St. E., P1 Level, Hamilton, Ontario L8N 1G6, Canada

## Abstract

**Background -:**

Environmental exposure to persistent organic pollutants (POPs) may lead to elevation of serum lipids, increasing risk of atherosclerosis with thromboembolism, a recognized cause of stroke. We tested the hypothesis that exposure to contaminants from residence near hazardous waste sites in New York State influences the occurrence of stroke.

**Methods -:**

The rates of stroke hospital discharges were compared among residents of zip codes containing hazardous waste sites with POPs, other pollutants or without any waste sites using information for 1993–2000 from the New York Statewide Planning and Research Cooperative System (SPARCS) database, containing the records of all discharge diagnoses for patients admitted to state-regulated hospitals.

**Results -:**

After adjustment for age and race, the hospitalization rate for stroke in zip codes with POPs-contaminated sites was 15% higher than in zip codes without any documented hazardous waste sites (RR 1.15, 95% CI, 1.05, 1.26). For ischemic stroke only, the RR was 1.17 (95% CI 1.04, 1.31). Residents of zip codes containing other waste sites showed a RR of 1.13 (95% CI, 1.02, 1.24) as compared to zip codes without an identified waste site.

**Conclusion -:**

These results suggest that living near a source of POPs contamination constitutes a risk of exposure and an increased risk of acquiring cerebrovascular disease. However further research with better control of individual risk factors and direct measurement of exposure is necessary for providing additional support for this hypothesis.

## Background

Cerebrovascular disease is a major public health problem [1]. In addition to well-documented modifiable risk factors of stroke, there is evidence for a link between a broad category of environmental factors and stroke, such as air pollution [2], environmental tobacco smoke [3], metals [4], pesticides [5], and other anthropogenic factors, including persistent organic pollutants (POPs).

POPs are chlorinated organic compounds [polychlorinated biphenyls (PCBs), dioxins and chlorinated pesticides] that are resistant to degradation and able to bio-accumulate in fatty tissues of living organisms. These compounds are semivolatile, and present in the atmosphere as vapors or adsorbed on suspended particles [6]. Multiple adverse health effects have been associated with the exposure to POPs [7,8] including cardiovascular pathologies, such as hypertension, ischemic heart disease, and atherosclerosis [9-11]. A major route of exposure to POPs is dietary, especially through the consumption of fish from contaminated waters. There is, however, growing evidence that suggests inhalation is a significant route of exposure to POPs and, especially, to PCBs [12,13]. Waste combustion and volatilization from wet soils or sewage sludge-amended land considerably contribute to elevation of PCB levels in the environment [14] and increase a possibility of human exposure through inhalation [15,16].

There is an increased incidence of some chronic diseases among individuals living near hazardous waste sites [17]. However, to our knowledge there have been no studies that explored an association between xenobiotic exposure from hazardous waste sites and cerebrovascular disease. We have performed a study using aggregate data to investigate the relationship between environmental exposure to POPs/PCBs and other wastes and hospital discharge rate of stroke among the New York State residents.

## Methods

SPARCS was used to obtain data on hospital discharge diagnosis of cerebrovascular disease among the New York State residents. SPARCS contains records of discharge diagnoses for all persons admitted as inpatients in all public and private New York hospitals, excluding federal-regulated facilities and mental health facilities. Non-hospitalized cases are not collected by the SPARCS registry. The database available to us contained the primary and up to 14 secondary discharge diagnoses in the format of International Classification of Disease, Ninth Revision (ICD-9), in addition to the zip code of residence, sex, age, and race/ethnicity of each patient. We have used the SPARCS data from 1993 to 2000.

Data on hazardous waste sites were obtained from the New York State Department of Environmental Conservation (NYSDEC). The major contaminants present and the zip code(s) for each site were extracted from the NYSDEC database. From a total of 818 State and Federal hazardous waste sites in New York State (excluding New York City), 396 sites contained POPs. These hazardous waste sites ("POPs") were located in 192 zip codes. Two hundred thirteen zip codes contained hazardous waste sites where the listed contaminants of concern did not include any POP, and these were categorized as "other waste". All of the other 994 zip codes, which contained no identified hazardous waste sites, were classified as "clean", although we recognize that these zip codes may contain wastes that have not been characterized. One subset of the "POPs sites" was examined separately: 78 zip codes along the PCB-contaminated portion of the Hudson River from Hudson Falls to New York City (30% of all people living in POPs-contaminated zip codes). Using the information from the Behavioral Risk Factor Surveillance System (BRFSS) we were able to compare behavior of the population along the Hudson River (available only at the level of counties, not zip codes) to the rest of New York State.

Demographic data on New York State residents (age, gender, race, and median household income for each zip code in Upstate New York) was obtained from Claritas Inc., an information resource company that provides information derived from the U.S. Census, with the zip codes used being the same as those used by the U.S. Postal Service.

The data from SPARCS, Claritas, and NYSDEC were merged on the basis of a zip code of residence to determine the rate of stroke hospital discharges among the individuals residing in three categories of zip codes ("POPs", "other waste", and "clean") for the years 1993–2000. Exposure was defined as a patient's residence in a zip code that contained or abutted at least one hazardous waste site. We used primary and all secondary ICD-9 codes 430 to 436 for cerebrovascular disease (with the fourth and fifth digits). Ischemic stroke was defined as codes 433.x1, 434.x1, and 436, while hemorrhagic cerebrovascular disease was identified as codes 430, 431, and 432.

We excluded all zip codes that were not constant from 1993 to 2000 and post office box zip codes (914 in total). Since New York City maintains its own hospitalization dataset and has unique sociodemographic characteristics, it was also excluded. After all the exclusions were made, 1399 zip codes remained in the study.

Finally, the analysis was restricted to White and African-American races because the numbers of Asians and Native Americans were small. We restricted analysis to patients between 25 to 64 years old in order to evaluate stroke frequency at an age at which stroke is relatively rare, expecting that that would provide a better indication of an elevation in risk should it exist.

### Statistical analysis

The stroke hospital discharge rate per 100,000 was calculated as the number of people discharged with cerebrovascular disease divided by the estimated total population. We used a Negative Binomial regression model, with the GENMOD procedure from SAS software. The Negative Binomial model was log linear: *Log *(*Expected Hospital Discharge Rate of Stroke*) = *log *(*total person-time*) + *β*_0 _+ *β*_1_*AGE5 + β_2_*AGE4 + β_3_*AGE3 + *β*_4_*GENDER + β_5_*RACE + β_6_*POPs sites + β_7_*Other waste, where "POPs" and "other waste" represented the exposure; age, gender, and race represented other dependent variables with a value of zero or one. Before formulating the final regression model, we assessed confounding by demographic variables (age, gender, and race). All statistical analyses were conducted using the SAS statistical software package, version 8.2 (SAS Institute, Inc., Cary, North Carolina).

The initial Negative Binomial regression analysis included all four quartiles of the median household income, estimated on a zip code level. However, the analysis of zip codes with the lowest and highest median incomes (the first and the fourth quartiles) showed the greatest population variability. Therefore, we restricted the Negative Binomial regression model to the middle-income zip codes (second and third quartiles), with the median household income ranging from $30,388.0 to $48,213.5.

## Results

**Figure 1:**
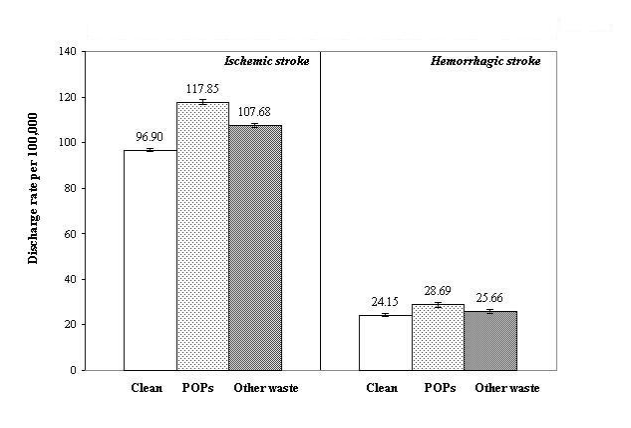
Crude hospital discharge rates for ischemic and hemorrhagic stroke in upstate New York, 1993–2000, for residents living in clean, POPs, and other waste site zip codes.

Figure 1 shows the crude rates of hospital discharge for ischemic and hemorrhagic stroke in the full dataset of 42,420,284 person-years over 1993–2000. For ischemic stroke there was a significantly greater number of discharges in residents of "POPs" sites as compared to either "clean" or "other waste" sites (P < 0.0001). For hemorrhagic stroke the elevation was significant in "POPs" sites as compared to "clean" sites (P = 0.003).

From 1993 to 2000 there were 28,216 stroke discharges in the three study zip code classes. Table 1 shows the population characteristics of the second and third income quartiles that were used for the further analysis. Such variables as age, gender, race and median income did not differ significantly among "POPs", "other waste" and "clean" zip codes. The age, gender and race distribution of individuals with stroke discharges and residing in the three groups of zip code are presented in Table 2.

**Table 1:** Population Characteristics*

	Person-years (%)		
	
	"POPs" zip codes	"Other waste" zip codes	"Clean" zip codes
Total† (16,880,516)	5,815,148 (34.5)	4,340,180 (25.7)	6,725,188 (39.8)
Average Income‡	$37,971.09	$36,547.13	$36,231.88
Age groups			
25–34	1,703,276 (29.3)	1,261,376 (29.1)	1,889,316 (28.1)
35–44	1,774,672 (30.5)	1,323,224 (30.5)	2,070,400 (30.8)
45–54	1,335,396 (23.0)	1,022,544 (23.6)	1,625,412 (24.2)
55–64	1,001,804 (17.2)	733,036 (16.9)	1,140,060 (16.9)
Gender			
Male	2,858,752 (49.2)	2,142,968 (49.4)	3,371,492 (50.1)
Female	2,956,396 (50.8)	2,197,212 (50.6)	3,353,696 (49.9)
Race			
White	5,361,748 (92.2)	3,934,472 (90.7)	6,425,104 (95.5)
African American	453,400 (7.8)	405,708 (9.3)	300,084 (4.5)

* This and all subsequent tables represent data for 1993–2000

† Total population is the total person-years from 1993 through 2000, which is the sum of the population in each category of zip codes each year for eight years.

‡ Average income was calculated as the sum of the median household income for each zip code divided by the number of zip codes in each category.

**Table 2:** Age Distribution for Stroke Discharges

	Stroke Discharges N (%)		
	
	"POPs" zip codes	"Other Waste" zip codes	"Clean" zip codes
Total Discharges*	10,220	7,506	10,490
Age groups			
25–34	322 (3.2†)	238 (3.2)	287 (2.7)
35–45	986 (9.6)	683 (9.1)	943 (9.0)
45–54	2,768 (27.1)	2,069 (27.5)	2,874 (27.4)
55–64	6,144 (60.1)	4,516 (60.2)	6,386 (60.9)

* The total stroke discharges in all three categories of zip codes was 28,216.

† The percentage of stroke discharges in the "POPs" sites in the age group 25–34 years.

The results of negative binomial regression are presented in Table 3. The rate ratio (RR) for stroke discharge in zip codes with POPs-contaminated hazardous waste sites was 1.15 (95% CI 1.05, 1.26) as compared to zip codes without any contamination. We also observed a RR of 1.13 (95% CI 1.02, 1.24) in "other waste" sites as compared to "clean" sites. As expected, age, race, and gender were significantly associated with the stroke discharges. The goodness-of-fit criteria demonstrated an adequate fitting of the model.

**Table 3:** Results of Negative Binomial Regression for All Types of Stroke

	Rate Ratio	95% Confidence Intervals for Rate Ratio	
Exposure			
"POPs" zip codes	1.15	1.05	1.26
"Other waste" zip codes	1.13	1.02	1.24
"Clean" zip codes*	1.00	1.00	1.00
Age Groups			
55–64	33.28	29.50	37.54
45–54	11.08	9.81	12.52
35–44	3.04	2.68	3.46
25–34*	1.00	1.00	1.00
Race			
African-American	2.06	1.90	2.24
White*	1.00	1.00	1.00
Gender			
Male	1.13	1.04	1.23
Female*	1.00	1.00	1.00

* Reference group

Table 4A and 4B show results of negative binomial regression for ischemic and hemorrhagic stroke. Rates for ischemic stroke were elevated in zip codes with sites containing POPs (RR 1.17, 95% CI 1.04, 1.31) and other waste (RR 1.14, 95% CI 1.01, 1.28), as compared to "clean" zip codes. For hemorrhagic stroke the RRs were not significantly different. Age, race, and gender were significantly associated with the hospital discharges of both types of stroke (data not shown).

**Table 4:** Negative binomial regression results for stroke, and comparison of Hudson River to "clean" zip codes.

Exposure	Rate Ratio	95% Confidence Interval	
**A. Ischemic Stroke**			
"POPs" zip codes	1.17	1.04	1.39
"Other waste" zip codes	1.14	1.01	1.28
"Clean" zip codes*	1.00	1.00	1.00
**B. Hemorrhagic Stroke**			
"POPs" zip codes	1.10	0.99	1.22
"Other waste" zip codes	1.04	0.93	1.16
"Clean" zip codes*	1.00	1.00	1.00
**C. Subset of Hudson River zip codes**†			
Hudson River zip codes	1.20	1.10	1.32
"Clean" zip codes*	1.00	1.00	1.00

* Zip codes with no identified hazardous waste sites (reference)

† Zip codes along the PCB-contaminated portion of the Hudson River

Table 4C shows the negative binomial regression model results for stroke for a subset of POPs zip codes, those along the Hudson River. As compared to "clean" zip codes, the RR was 1.20 (95% CI 1.10, 1.32). This 20% elevation of stroke diagnosis in Hudson River zip codes indicates that the relationship between residential exposure to POPs and the rate of stroke discharges is similar for the people living along the Hudson River and general population of Upstate New York.

## Discussion

Our results suggest that living in zip codes that contain hazardous waste sites is associated with an increased rate of hospital discharges for stroke, especially ischemic stroke. The regression model, when limited to middle income, showed a 15% elevation of hospital discharge rates for stroke in zip codes with POPs waste sites, independent from patient's age, race, or gender even though increased age, being male and being African-American were all significant but independent risk factors. In POPs-contaminated zip codes along the Hudson River the increase of hospital discharge rates for stroke was even larger. However, as previously reported [18] individuals along the Hudson River on average have higher income, exercise more frequently, consume more fruits and vegetables, and there are more non-smokers and former smokers among them than among the people from the rest of Upstate New York. Therefore, these known risk factors alone cannot fully explain the observed geographical variation of stroke discharge rates. This provides some additional support for the hypothesis that exposure to POPs is a potential contributing factor. If the current data reflect true associations, it is unlikely that fish consumption, usually considered to be the primary route of exposure, is the only important one. Sport fishing and fish-eating habits are not defined by zip code of residency. Inhalation of POPs in the vapor phase or bound to particulates, or ingestion of POPs-contaminated particulates with foodstuff is the most likely exposure pathway.

The stroke discharge rates in zip codes that have a hazardous waste site, but not one with POPs, were also found to be elevated (12%) after the negative binomial regression analysis. However, the substantial heterogeneity in this group of pollutants (heavy metals, volatile organic substances, radiation, etc.) and existence of toxicological/biological interactions among them prevents us from drawing definite conclusions about their influence on stroke occurrence.

The results of this study are consistent with the findings of previous investigations that show elevations in various diseases in residents living near hazardous waste sites [2,13,17-19]. We [19] have reported an elevation is hospitalization for coronary artery disease and myocardial infarction using SPARCS data and the same characterization of zip codes as applied in this study. We found that residence in a POPs zip code in all of upstate New York resulted in a 15% elevated rate of hospitalization for coronary artery disease, and a 20% elevation in acute myocardial infarction. In the subset of POPs zip codes along the Hudson River the rates were elevated by 35.8% for coronary artery disease and by 39.1% for acute myocardial infarction.

Stroke has many pathologic factors similar to those in cardiovascular disorders. Ischemic stroke is a "brain attack" and its etiology is similar to that of myocardial infarction [20], while hemorrhagic stroke is often secondary to hypertension. Several studies have reported that exposure to POPs/PCBs is associated with elevated frequency of several chronic diseases, such as ischemic heart disease, diabetes, hypertension and chronic liver disease [9,21,22]. One mechanism linking exposure to POPs and cardiovascular diseases may be through increased serum lipids, a known risk factor for atherosclerosis. Serum lipids [23], plasma triglyceride levels [24], and total cholesterol [11] are elevated in dioxin and PCB-exposed populations. Monkeys exposed to dioxin and PCBs showed a 3- to 5-fold elevation in serum triglyceride concentrations [25], and similar effects have been reported in female rats after exposure to PCBs [26]. Dioxin and coplanar PCBs cause the production of reactive oxygen species which, in turn, cause damage to endothelial cells and promote the formation of foam cells and atherosclerotic plaques [27]. The combination of elevated serum lipids in the presence of damaged endothelial cells would contribute to the risk of development of cardiovascular pathology and cerebrovascular disease, especially ischemic stroke. Indeed, our results (Table 4A) show a stronger relation with ischemic than hemorrhagic stroke.

We have previously reported an elevation in hospital discharges for infectious respiratory disease in "POPs" zip code residents and compared to "clean" and "other waste" zip codes [18]. As in the present study, we found that the relationships were not reduced in zip codes along the Hudson River where income is higher and smoking rates lower than in the rest of New York State. Using a different data set, the New York State birth registry, we have also found an elevation in low birth weight in children born to residents of zip codes containing a PCB contaminated site [11]. In sum, together with our recent report on coronary heart disease and myocardial infarction [19], these results with four different health outcomes and two different datasets provide support for the hypothesis that residence near POPs sites increases risk of several types of disease.

There are clear limitations in determining cause and effect in partially ecologic study designs such as we have used. Given that the study was based on aggregative data, we do not have a direct measure of exposure (a zip code of residency is a crude surrogate for exposure assessment), and have no information on the duration of individual residence in each specific zip code. It is possible, and indeed likely, that some individuals residing in a POPs zip code were not exposed because of short length of residence, or residence still in the zip code but far from the hazardous waste site. The information on income was available only at a zip code level, which allowed marginal adjustment for socio-economic status. It does not adjust for the range of income in any one zip code, nor the possibility that within a zip code the income is less among those living closer to the waste sites. Therefore, it is possible that the observed associations were influenced by some underdetermined factors (e.g. access to health care) and might not be applicable to every socioeconomic subpopulation.

There are many other potential sources of confounding with the groups, and these are only partially controlled for by use of the BRFSS for the Hudson River population. BRFSS information is currently available only at a county level, not at the zip code level. While the BRFSS provides information on average behaviors within a county, it is still possible that those individuals who experience strokes differ from this average.

While the absence of personal identifiers restricted our ability to account for some potential confounders/effect modifiers, hospitalization data obtained in a mandatory manner for a number of years, because of the large numbers involved, has considerable potential for generating and testing hypotheses regarding the causes of disease [28]. In spite of these limitations our observations provide support for the general hypothesis that living near hazardous waste sites poses risk of exposure and of disease.

## Conclusion

We found a statistically significant elevation of hospital discharge rates for stroke in zip codes with POPs-contaminated hazardous waste sites, and to a lesser degree with "other waste" sites, when compared to zip codes that do not have any identified waste sites. These observations suggest that living near a waste site contaminated with POPs is associated with the risk of inhalational and/or ingestional exposure and an increased risk of acquiring cerebrovascular disease. Further research involving control for individual risk factors and direct exposure assessment techniques is necessary for providing additional evidence for this hypothesis.

## List of Abbreviations

BRFSS – Behavioral Risk Factor Surveillance System

ICD-9 – International Classification of Disease, Ninth Revision

NYSDEC – New York State Department of Environmental Conservation

PCBs – polychlorinated biphenyls

POPs – persistent organic pollutants

RR – rate ratio

SPARCS – New York Statewide Planning and Research Cooperative System

## Competing interests

The author(s) declare that they have no competing interests.

## Authors' contributions

IS performed the data analysis as a requirement of his MPH program, performed the statistical analysis, and wrote the first draft of the paper. XH coordinated the use of the SPARCS and hazardous waste datasets. LL provided overall statistical direction for the study. DOC designed the study and supervised the data analysis. All authors read and approved the final manuscript.
